# Interventional inflammatory bowel disease: endoscopic therapy of complications of Crohn’s disease

**DOI:** 10.1093/gastro/goac045

**Published:** 2022-09-14

**Authors:** Bo Shen

**Affiliations:** Center for Inflammatory Bowel Disease, Columbia University Irving Medical Center/New York-Presbyterian Hospital, New York, NY, USA

**Keywords:** Crohn’s disease, complication, balloon dilation, endoscopy, fistula, fistulotomy, sinusotomy, stricture, strictureplasty, stricturotomy, therapy

## Abstract

Endoscopic therapy for inflammatory bowel diseases (IBD) or IBD surgery-associated complications or namely interventional IBD has become the main treatment modality for Crohn’s disease, bridging medical and surgical treatments. Currently, the main applications of interventional IBD are (i) strictures; (ii) fistulas and abscesses; (iii) bleeding lesions, bezoars, foreign bodies, and polyps; (iv) post-operative complications such as acute and chronic anastomotic leaks; and (v) colitis-associated neoplasia. The endoscopic treatment modalities include balloon dilation, stricturotomy, strictureplasty, fistulotomy, incision and drainage (of fistula and abscess), sinusotomy, septectomy, banding ligation, clipping, polypectomy, endoscopic mucosal resection, and endoscopic submucosal dissection. The field of interventional IBD is evolving with a better understanding of the underlying disease process, advances in endoscopic technology, and interest and proper training of next-generation IBD interventionalists.

## Introduction

Crohn’s disease (CD) is a primary phenotype of inflammatory bowel disease (IBD). The majority of patients with CD eventually develop strictures, fistulas, and abscesses, and some of them may develop colitis-associated neoplasia (CAN) [1, 2]. CD is classified as non-stricturing/non-penetrating (B1), stricturing (B2), or penetrating (B3), based on the disease behavior [3]. The cumulative risk for the development strictures or fistulas was reported from 34%–52% at 5 years and 40%–70% at 10 years after diagnosis [4–6].

The main management strategies for CD are medical, endoscopic, and surgical treatment. While medical therapy plays a key role in the treatment of B1 disease and inflammatory components of B2 or B3 disease, and perianal disease, endoscopic and surgical therapy are the main treatment modalities for structural or neoplastic complications. The inflammatory component of strictures may benefit from medical therapy, particularly biologics [7]. Patients with stricturing CD or bowel obstruction were excluded in nearly all published randomized–controlled trials of biologics [8]. The role of biological therapy or small-molecule therapy in penetrating CD is described in the treatment of perianal disease or enterocutaneous fistula. In contrast, the application of endoscopic therapy can be extended to penetrating CD or CD surgery-associated acute or chronic anastomotic leaks, perianal disease, enterocutaneous fistula, and enteroenteric fistula. In addition, advanced endoscopic treatment modalities, such as endoscopic mucosal resection (EMR) and endoscopic submucosal dissection (ESD), have been explored in CAN.

Surgical interventions including bowel resection with anastomosis and stricturoplasty are discussed in a separate article in this issue. In the review, indications and basic techniques of interventional IBD are discussed.

## Strictures

**Figure 1.**
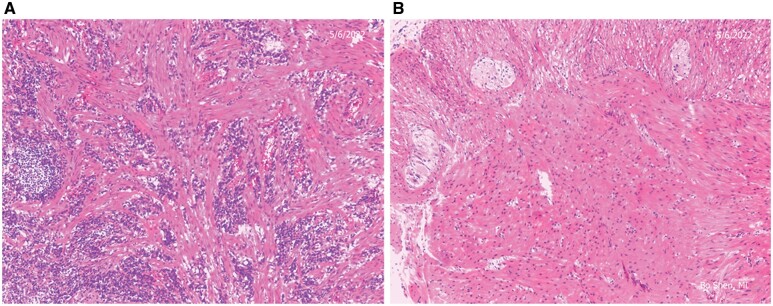
Histopathology of intramural stricture. (A) Fibrosis and infiltration of inflammatory cells (10X, H&E stain); (B) muscular hypertrophy neuronal hyperplasia (10X, H&E stain).

**Figure 2.**
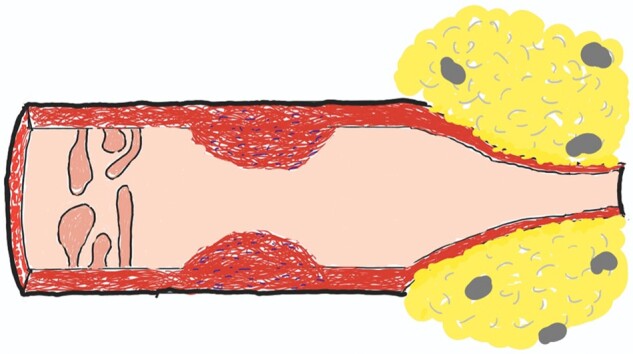
Three types of strictures—intraluminal (inflammatory pedunculated polyps; left), intramural muscular hypertrophy and fibrosis (center), and extraluminal constriction by creeping fat and lymph nodes (right)

A stricture is defined as the narrowing of the intestinal lumen. On histology, intramural stricture consists of fibroblasts, collagens, intervening acute or chronic inflammatory cells, hypertrophy of muscle fibers, and neuronal hyperplasia (Figure 1). In addition, intraluminal (such as bezoars and pedunculated inflammatory polyps) and extraluminal (such as creeping fat and adhesion) factors contribute to the luminal narrowing in CD (Table 1 and Figure 2). A concept of constrictive stricture from extraluminal factors in CD has been described [9].

**Table 1.** Contributors for bowel lumen stenosis and preferred therapy

Factors	Preferred first-line therapy
Intraluminal	
Bezoars and foreign bodies	Endoscopic therapy
Polyps	Endoscopic therapy
Prolapse	Endoscopic therapy
Intramural	
Fibrosis	Endoscopic therapy
Muscle	Medical and endoscopic therapy
Neuronal	Medical and endoscopic therapy
Extraluminal	
Creeping fat	Medical and surgical therapy
Adhesions	Surgical therapy
Mass	Surgical therapy

Intramural strictures are categorized into inflammatory, fibrostenotic, and mixed types, by endoscopy, histology, and cross-sectional imaging. Strictures can further be classified into: (i) primary vs anastomotic; (ii) single vs multiple; (iii) short vs long with a cut-off length of 4–5 cm; (iv) benign vs malignant; (v) ulcerated vs non-ulcerated; (vi) stricture with or without prestenotic dilation; and (vii) simple or complex (e.g. those with associated fistulas or abscesses) [10].

The main endoscopic treatment modalities for strictures are endoscopic balloon dilation (EBD), endoscopic stricturotomy (ESt), endoscopic strictureplasty (ESTx), and endoscopic stenting. Most short (<4–5 cm) primary or anastomotic strictures in CD are amenable to endoscopic therapy. The consensus guidelines suggested the use of an immediate passage of endoscope after treatment as technical success and surgery-free survival as long-outcome [10]. The main adverse outcomes are procedure-associated bleeding and perforation. While most patients with strictures, especially primary CD strictures, eventually require surgical intervention, some patients undergoing endoscopic therapy are able to avoid surgery or defer surgery. The pacing-out of the need for surgical intervention is particularly useful in those who would otherwise have multiple surgeries. Therefore, the main goals of endoscopic therapy in CD strictures are avoidance or increase in interval of surgical intervention. The choice of EBD, ESt, ESTx, or stenting is based on the general medical condition of patients, nature of the disease process, characteristics of strictures, and expertise of the endoscopist. Pre-procedural abdominal and pelvic imaging, such as computed tomography, magnetic resonance imaging, and gastrograffin enema via stoma, fistula, or anus should be performed to characterize the location, number, degree, length, and associated conditions of strictures. Intraprocedural fluoroscopic guidance may provide additional information.

### Association between strictures and fistulas

**Figure 3.**
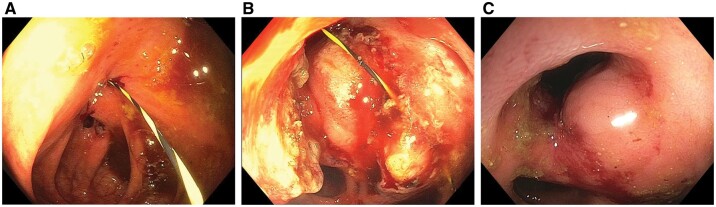
Ileocecal valve stricture and fistula. (A) Ileocecal fistula caused by long-standing ileocecal valve stricture; (B) endoscopic valvectomy/stricturotomy/fistulotomy over a guide wire; (C) follow-up colonoscopy showed patent ileocecal valve with disappearance of the ileocecal fistula.

A common pathway of phenotypic evolution in small bowel CD is characterized by chronic transmural inflammation, stricture with gradual prestenotic luminal dilation, and formation of fistula proximal to strictured bowel lumen. Classical examples are ileocecal fistula with a stricture at the ileocecal valve (Figure 3A), ileosigmoid fistula with a stricture at the distal ileum, and colo-duodenal fistula with a stricture at the hepatic flexure. Some short enteroenteric fistula with concurrent stricture, e.g. ileocecal fistula and ileocecal valve stricture, may be treated with endoscopic fistulotomy (Figure 3).

**Figure 4.**
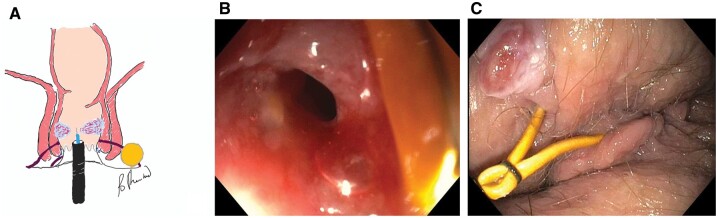
Association between anorectal stricture and perianal fistulas. (A) Endoscopic stricturotomy of anorectal ring stricture with perianal fistulas distally; (B) the tight anorectal stricture; (C) perianal fistulas with a seton distally.

The disease course of fibrostenotic and perianal CD is different from the small bowel counterparts. Often we encounter perianal fistulae and abscesses with tight anal strictures at the anorectal ring, just proximal to the origin of the perianal fistula (Figure 4A).

### EBD

**Figure 5.**
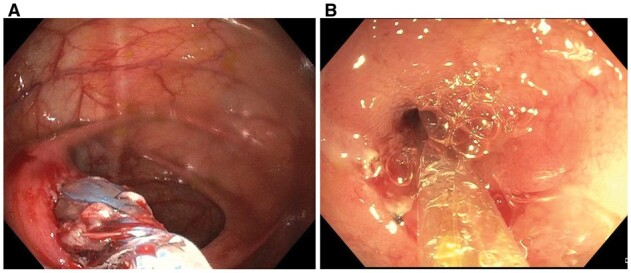
Endoscopic balloon dilation of strictures. (A) Balloon dilation of primary stricture at the ileocecal valve; (B) balloon dilation of end-to-side ileocolonic anastomosis stricture.

EBD has been applied to strictures at any location of the gastrointestinal (GI) tract and is increasingly used in the treatment of primary and anastomotic strictures in CD (Figure 5). Performance of EBD is less technically demanding than ESt, ESTx, or endoscopic stenting. Most gastroenterologists and some GI surgeons feel comfortable performing EBD. EBD can be performed in an antegrade or retrograde fashion with an ultimate targeted balloon size of 18–20 mm. The consensus guidelines did not recommend the routine intralesional injection of long-acting steroids during or before EBD or dependently intralesional injection of antitumor necrosis factor (TNF) agents [10].

Technical success and long-term outcomes of EBD have been extensively studied with small and large case series. It is estimated that the technical success of EBD in the treatment of primary or anastomotic strictures of CD is ∼85%–95% and two-thirds of patients were about to avoid surgery during the follow-up periods [8]. Reported frequency of bleeding and perforation was ∼1%–5% [8].

The outcome of EBD in the treatment of primary CD strictures or strictures of ileocolonic anastomosis has been compared with surgical resection and re-anastomosis. As expected, surgical resection was shown to be more effective than EBD in terms of the surgery-free survival, but of higher risk for adverse events [11, 12]. However, periodic EBD of ileocecal valve strictures deferred 6.45 years for the subsequent surgery [11]. The surgery-free survival curves between EBD and surgical resections separated more in the treatment of anastomotic stricture without prestenotic dilation than those without prestenotic dilation [11]; and separated more in primary CD strictures than in the treatment of anastomotic strictures in CD [12]. Controlled studies are needed to compare outcomes between EBD and surgical strictureplasty in CD strictures.

In this author’s clinical practice, tissue biopsy of the stricture should be taken to rule out malignancy at the index endoscopy and yearly afterward. EBD is performed in patients with short (<4–5 cm), straight, inflammatory, or mixed inflammatory and fibrotic primary or anastomotic strictures. The targeted balloon size is 18–20 mm, which may require graded dilation or dilation with multiple sessions. The consensus guideline from the Global Interventional IBD Group did not recommend an intralesional injection of long-acting steroids after or during EBD [10]. Most patients require periodic endoscopic intervention to keep the patency of the lumen. If the patients require EBD more often than every 3–12 months, alternative endoscopic approaches, such as ESt and ESTx, or surgical intervention are recommended.

### Endoscopic stricturotomy and strictureplasty

Endoscopic electroincision of strictures has been reported for the treatment of non-IBD strictures in the upper GI tract. The role of endoscopic electroincision with ESt or ESTx has recently been explored by the author’s group. The consensus guidelines from the Global Interventional IBD Group recommended ESt being a preferred endoscopic approach for anorectal strictures [10]. This author’s group has routinely performed ESt for severe anorectal or anopouch stricture with a great outcome and ignorable risk of complications (Figure 4) [13].

**Figure 6.**
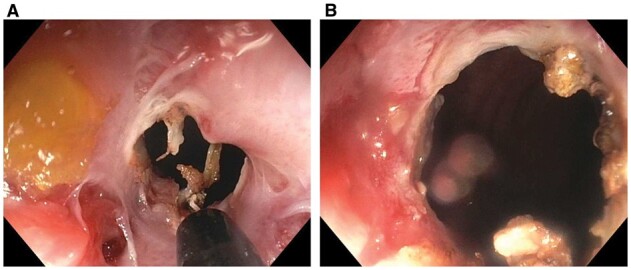
Endoscopic stricturotomy. (A) Fibrotic primary anorectal stricture undergoing endoscopic stricturotomy with an insulated-tip knife; (B) post-procedural appearance of the treated stricture.

**Figure 7.**
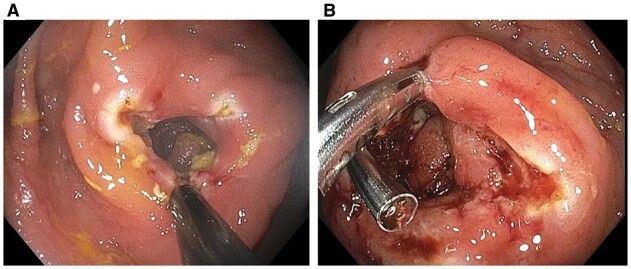
Endoscopic strictureplasty. (A) Stricture at the end-to-side ileocolonic anastomosis being treated with endoscopic stricturotomy; (B) placement of the endoclips in the radially incised stricture as spacers, which turns stricturotomy to strictureplasty.

In comparison to EBD in which strictured tissue is displaced, ESt involves excision of the tissue and ESTx incision and spacing of the tissues. ESt or ESTx has been shown to be more effective than EBD with a lower risk of perforation but carries a higher risk of late-onset bleeding than EBD [14–16]. The choice between ESt, ESTx, and EBD is determined by inflammatory composition, severity, length, and location of strictures. While EBD can be performed in inflammatory strictures, ESt or ESTx is performed in short (<4–5 cm) fibrotic strictures. ESt is particularly useful in the treatment of anorectal strictures [13]. Circumferential ESt of the posterior wall of the anorectal or anopouch strictures can avoid iatrogenic injury to the anal sphincter or vagina (Figure 6). Therefore ESt is recommended as first-line therapy for the treatment of anorectal or anopouch strictures [10]. Since anorectal stricture often coexists with perianal fistulas and abscesses, effective endoscopic treatment of the stricture along with medical therapy may favorably taper the course of perianal disease. ESTx is often effective in the treatment of strictures of length 1–2 cm at the ileocolonic anastomosis (Figure 7). Patients with refractory strictures to EBD or electroincision or strictures at the pylorus or ileocecal valve may be treated with a combined EBD and ESt or ESTx.

### Endoscopic stenting

**Figure 8.**
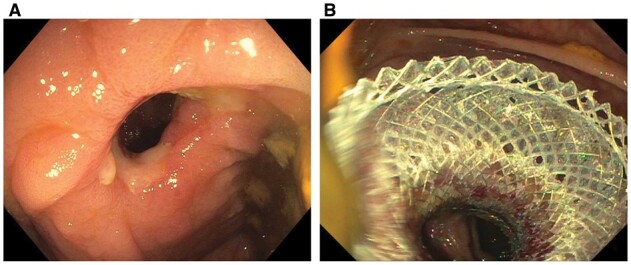
Endoscopic placement of a lumen-apposing stent. (A) Side-to-side ileocolonic anastomosis stricture that was previously treated with balloon dilation and endoscopic stricturotomy. Both procedures resulted in excessive bleeding. (B) A lumen-apposing stent was placed across the 1-cm-long stricture.

Previous case reports or small case series suggest that some IBD strictures may be treated with self-expandable metal stents [17–19]. A recent randomized–controlled trial (*n *=* *80) showed that endoscopic stenting was less effective and carried a higher risk of adverse effects than EBD [20]. A separate small randomized–controlled trial (*n *=* *14), however, showed that endoscopic stent was more effective than EBD [21]. The most technical challenge of endoscopic stenting is stent migration, even with the use of endoclips for security. In this author’s experience, endoscopic stenting is amenable for the treatment of stricture with marked prestenotic luminal dilation. The latter may serve as a shoulder for the security of the proximal end of the self-expandable metallic stent. Lumen-apposing stent may be used for tight and short side-to-side stricture of ileocolonic anastomosis (Figure 8). However, the role of endoscopic stenting in the treatment of primary or anastomotic stricture needs to be further defined.

### Fistulas and abscesses

Penetrating CD is characterized by the formation of fistulas and abscesses in the form of enteroenteric fistula, enterocutaneous fistulas, enterobladder fistulas, perianal fistulas, rectovaginal fistulas, and pouch-vaginal fistulas, and intestine and bladder. The pathogenesis of fistula in CD is poorly understood [22–25]. The fistula in CD can originate from the native diseased bowel or be attributed to chronic anastomotic leaks. The formation of fistula and stricture is closely associated. Intestinal fistula or abscess often coexists with downstream strictures, while perianal fistulas commonly have strictures at the anorectal ring proximately. In this author’s experience, ESt and ESTx of strictures in selected patients may enhance the efficacy of medical therapy of CD-associated enteroenteric fistula (Figure 3) or perianal fistula (Figure 6).

### Endoscopic fistulotomy

Short, shallow CD fistulas of the ileocecal or perianal areas may be treated with endoscopic fistulotomy. Ileocecal fistulas often result from a long-standing stricture at the ileocecal valve or terminal ileum. Endoscopic fistulotomy or complete valvectomy can be safely performed (Figure 3) [26]. Endoscopic fistulotomy in this setting may enhance the efficacy of medical therapy on the inflammation at the terminal ileum proximal to the ileocecal valve stricture and ileocecal stricture [27]. Some enteroenteric fistula from chronic suture-line or staple-line leaks of bowel surgery in IBD may also be treated with endoscopic fistulotomy [26].

### Endoscopic incision and drainage

Incision and drainage, and endoscopy-guided seton placement can be performed in CD-associated perianal fistulas and abscesses in CD [28–30]. Transluminal drainage stent or catheter placement through the primary orifice of enteroenteric fistula, enterocutaneous fistula, or bowel-fistula-associated abscess resulting from the *de novo* CD process is not recommended. However, placement of a drainage catheter or stent can be attempted via an anastomotic fistula-associated abdominal or pelvic abscess if transcutaneous drainage via interventional radiology is not feasible.

### Endoscopic closure

Endoscopic closure of bowel wall defects is a routine clinical practice in gastroenterology. Commonly used tools are through-the-scope clips (TTSC), over-the-scope clips (OTSC), and suturing devices. Some endoscopists have been tempted to use OTSC to close the primary orifice of Crohn’s fistula [31]. The main goal of endoscopic therapy for the fistula is to permanently or temporarily close the primary orifice of the fistula tract, promoting healing of enteroenteric fistulas, rectovaginal fistulas, or pouch-vaginal fistulas with combined medical therapy. The reality of endoscopic closure of the primary orifice of a CD fistula along the bowel wall is the lack of long-term efficacy [28].

## Lumen-blocking materials and lesions and bleeding

**Figure 9.**
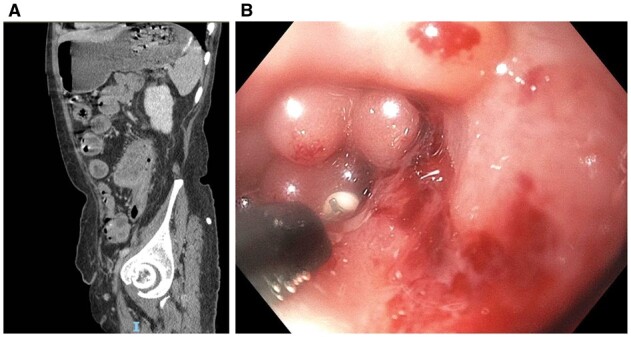
Removal of lumen-blocking inflammatory polyps. (A) Large intraluminal polyps in the descending colon on computed tomography; (B) endoscopic polypectomy.

**Figure 10.**
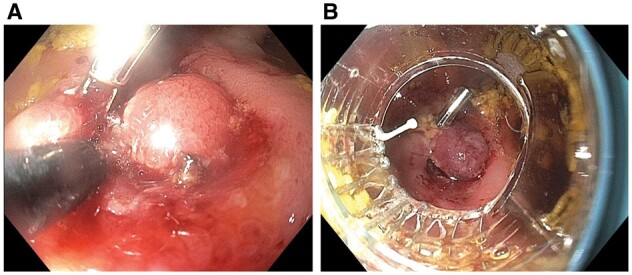
Endoscopic electroincision and banding of anastomotic stricture with proximal bowel prolapse. (A) Endoscopic stricturotomy of ileosigmoid anastomosis stricture; (B) endoscopic band ligation of prolapse neo-terminal ileum across the treated stricture.

Patients with CD can have lumen-blocking materials or lesions in the presence or absence of intramural strictures (Table 1). Intramural strictures, extraluminal constriction or compression, GI dysmotility, and surgery-altered bowel anatomy (such as anastomosis and surgical strictureplasty) put patients with CD at risk for the retention of bezoars, capsules, or foreign bodies, or development of inflammatory polyps or mucosal prolapse. The patients often present with symptoms of partial bowel obstruction, bleeding, or anemia. Endoscopic retrieval of bezoars and foreign bodies (including retained capsule endoscopes) can be safely performed. Concomitant endoscopic therapy of strictures with EBD, ESt, ESTx, or endoscopic laser fragmentation of calcified bezoars may be needed [32]. The lumen-blocking inflammatory polyps or mucosal prolapse can be treated with endoscopic polypectomy (Figure 9) or banding ligation (Figure 10).

## Post-operative complications

Post-operative complications in CD are common. The common complications are anastomotic bleeding, anastomotic strictures, and acute or chronic anastomotic leaks [33]. The endoscopist should be familiar with post-operative bowel anatomy by careful review of operative reports, pre-procedural imaging, and prior endoscopy report before delivering endoscopic therapy [10, 34].

### Anastomotic bleeding

**Figure 11.**
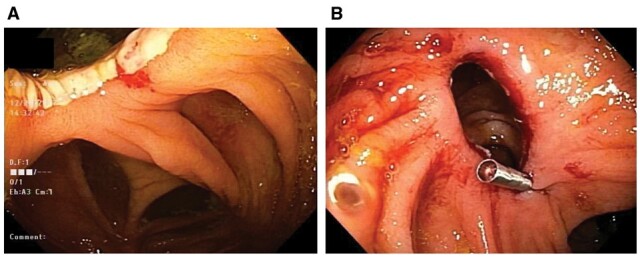
Endoscopic treatment of anastomosis bleeding. (A) Bleeding at a side-to-side ileocolonic anastomosis; (B) the bleeding was controlled by the placement of a hemoclip.

Bleeding from anastomosis can occur anytime post-operatively, although it is not common or is under-recognized [35]. Purported risk factors include surgical ischemia, the use of non-steroidal anti-inflammatory drugs or antiplatelets, and the underlying active CD. Acute or chronic anastomotic bleeding is preferred to be controlled by endoscopic management. There has been concern about the risk of anastomosis separation by immediate (<1–2 weeks) post-operative endoscopy. However, careful endoscopy with minimal air insufflation can safely be performed. This author has used topical spray or injection of hypertonic glucose and endoscopic clipping to control anastomotic bleeding (Figure 11).

### Prolonged post-operative ileus

Prolonged (>3 days) post-operative ileus after bowel resection surgery in CD is common [36]. The prolonged post-operative ileus can present forms of upper and lower GI ileus. Nasogastric tube has been standard practice. Decompression tube or nasogastric tube placed via the anus for post-operative or post-procedural ileus has been used in selected patients.

### Anastomotic strictures

In post-operative CD, common locations of anastomotic strictures following bowel resection and anastomosis and strictureplasty are the ileocolonic anastomosis, ileorectal anastomosis, enteroenteric anastomosis, and the inlet or outlet of the strictureplasty site. Anastomotic strictures usually develop over time. It is believed that most anastomotic inflammation results from underlying CD, rather than surgical ischemia, based on a histopathological study [37]. Whether ileocolonic anastomosis strictures stem mainly from the underlying CD rather than ischemia is not known.

**Figure 12.**
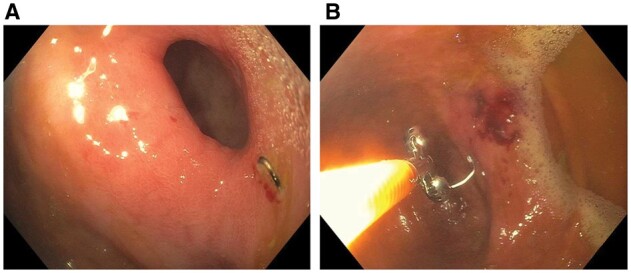
Endoscopic removal of staples at the anastomosis. (A) A dislodged staple at an ileorectal anastomosis stricture that was considered a contributing factor to bleeding and stricture; (B) endoscopic removal of staples by biopsy forceps.

Anastomotic strictures in CD can safely be treated with EBD, ESt, or ESTx (Figures 5 and 7) [10–12, 38–40]. Anastomotic strictures refractory to the endoscopic therapy should be evaluated for prolapse of the bowel proximal to the treated anastomosis (Figure 10) or presence of extraluminal constriction or compression. Removal of dislodged staples along the anastomosis may improve the outcome of endoscopic treatment (Figure 12). Configuration of anastomosis, strictureplasty, and stoma can pose challenges to endoscopic treatment of strictures.

**Figure 13.**
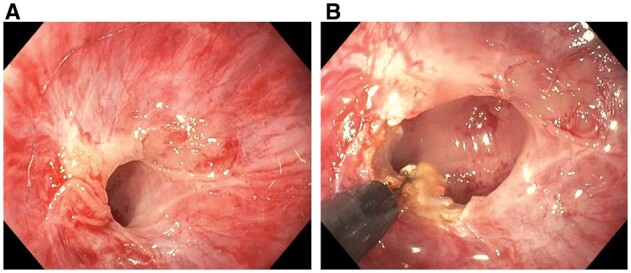
Endoscopic stricturotomy of diversion-associated distal rectal stricture in Crohn’s disease. (A) A severely friable stricture at the distal rectum; (B) endoscopic stricturotomy with an insulated-tip knife.

Patients who have ileostomy or colostomy for refractory CD commonly develop complications at the stoma or area around the stoma. Among them, strictures at the skin and fascia level can be appreciated by a careful ileostomy or colonoscopy via the stoma. These strictures can also be treated with EBD or ESt [41]. In addition, longer fecal diversion can result in stricture formation at the distal bowel or anorectal area. Diversion-associated strictures and proximal bowel are friable, posing a risk of bleeding and perforation with EBD or bougie dilation. Therefore, endoscopic ESt is preferred for the treatment of diversion-associated strictures (Figure 13).

### Acute anastomotic leaks

Patients with CD having bowel resection are more at risk for the development of anastomotic leaks than those without IBD. The reported risk factors include use of corticosteroids [42, 43], smoking [44], anemia [44, 45], blood transfusion [45, 46], hypoalbuminemia [44, 45], stricturing or fistulizing diseases [47], larger mesenteric fat area [48], end-to-end anastomosis (as compared to side-to-side anastomosis) [49], anastomotic tension [50], and repeat resection and anastomosis [51]. The negative impact of perioperative use anti-TNF biologics [44, 52, 53] and histologic resection margin on post-operative infectious complications has been controversial [44, 45, 54].

**Figure 14.**
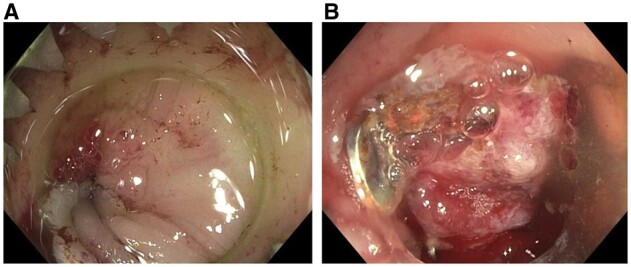
Endoscopic closure of acute anastomotic leak. (A) Fresh ileocolonic anastomosis leak with insertion of an anchor; (B) deployment of an over-the-scope clip at the leak.

The endoscopic treatment modalities for acute anastomotic leaks include vacuum pressure therapy, endoscopic TTSC or OTSC, fibrin glue injection, and stenting [55–60]. Acute fresh anastomotic leaks in <1–2 weeks respond favorably to the early endoscopic intervention (Figure 14). Minimal air insufflation is the key to avoiding exacerbating anastomotic leaks. Endoscopic treatment is more feasible in those with proximal fecal diversion and stoma in place. In acute fresh anastomotic leak, endoscopic pre-clipping tissue debridement of the anastomosis site is not necessary.

Acute anastomotic leaks usually lead to adjacent abscesses or sepses. Endoscopic drainage with a stent or catheter may be attempted if surgical or radiological drainage is not feasible.

### Chronic anastomotic leaks

**Figure 15.**
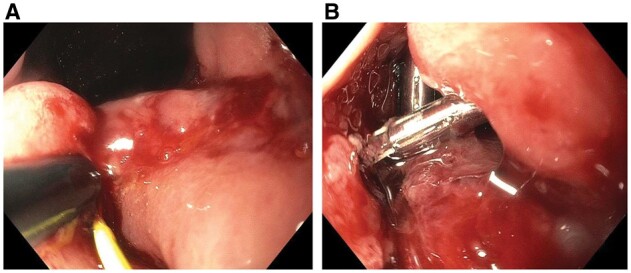
Endoscopic fistulotomy of chronic anastomosis leak in Crohn’s disease of the pouch. (A) Endoscopic fistulotomy along a soft-tip guide wire; (B) placement of endoclips to keep the treated fistula track open.

Chronic anastomotic leaks commonly present with fistulas, sinuses, or chronic abscess cavities. Endoscopic treatment strategies for chronic anastomotic leaks are different from those for acute anastomotic leaks. Preferred definitive endoscopic therapy modalities of chronic anastomotic leaks are fistulotomy (Figure 15) or sinusotomy in eligible patients, such as those with short anastomotic leak-associated enteroenteric fistula [26] and presacral sinus [61–63].

Endoscopic clipping may be performed as an alternative. Pre-clipping tissue debridement with cytology brush or argon plasma coagulation is required to enhance the efficacy of endoscopic closure with TTSC or OTSC [64].

## Neoplasia of small and large bowel and perianal region

Patients with persistent or chronic inflammation of the large bowel from IBD are at risk for the development of CAN [65–67]. Patients with Crohn’s colitis carry a similar risk for CAN to those with ulcerative colitis (UC) [68, 69]. As in UC, Crohn’s colitis with primary sclerosing cholangitis (PSC) is associated with a higher risk of CAN than Crohn’s colitis without primary sclerosing cholangitis [70]. High-definition white-light colonoscopy, dye-based chromocolonoscopy, and virtual chromocolonoscopy are recommended for surveillance. In addition, small bowel adenocarcinoma can develop in patients with long-standing CD [71]. Occasionally adenocarcinoma can develop at the entero-enteric, ileocolonic-, colo-colonic, colorectal, or colon–anal anastomoses. Patients with CD-associated chronic perianal fistulas or anorectal strictures are at risk of neoplasia of glandular or squamous cell source [72]. The consensus document from the Global Interventional IBD Group recommended routine surveillance endoscopy in these patients [10]. Therefore, a surveillance biopsy should be taken at index endoscopy in all CD patients undergoing endoscopic therapy and subsequent surveillance yearly.

**Figure 16.**
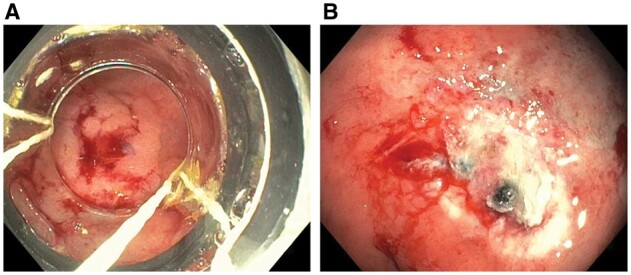
Endoscopic mucosal resection of colitis-associated dysplasia. (A) A slightly raised dysplastic lesion of the rectum in a patient with long-standing ulcerative colitis treated with endoscopic mucosal resection; (B) post-resectional endoscopic appearance.

CAN in CD can have endoscopic presentations, such as polypoid, raised, flat, or decompressed lesions, similar to those in UC. In addition, CAN may present with colonic strictures. As in CAN in UC [73–75], discrete, polypoid, or raised lesions may be removed by polypectomy, EMR (Figure 16), or ESD, although the literature on the endoscopic therapy of neoplastic lesions in CD is limited [76]. Long-term oncological outcomes of endoscopic therapy of IBD-associated IBD remain to be further defined.

Endoscopic excisional therapy of strictures with neoplasia is not recommended. Rather, bowel resection is warranted. The role of endoscopic treatment of neoplastic lesions of glandular or squamous source in perianal or anorectal CD has not been investigated. Topical injection to perianal fistulas in CD with stem cells or stromal cells has extensively been studied and in some parts of the world cell therapy has been a part of routine clinical practice [77, 78]. It is not clear whether the cell therapy has a beneficial or adverse impact on the development of perianal neoplasia.

## Summary

The evolving field of interventional IBD currently covers endoscopic treatment of strictures; fistulas and abscesses; lumen-blocking bezoars, foreign bodies, or inflammatory polyps, and bleeding; IBD surgery-associated complications; and CAN (Table 2). The main goals of endoscopic therapy are to relieve symptoms; improve patients’ quality of life; reduce the risk for the development of further adverse consequences of the complications; complement medical and surgical treatment; defer or avoid the need for surgery; and save healthcare-related costs. There is room for improvement in techniques, technology, equipment, and supplies, along with a better understanding of the pathogenesis of the structural complications of IBD.

**Table 2.** Current indications for interventional inflammatory bowel disease

Indication	Method
Strictures	Balloon dilation Stricturotomy/strictureplasty Stenting
Fistulas and abscesses	Fistulotomy Stenting Incision and drainage
Bezoar, foreign bodies, other blocking luminal lesions; bleeding; fecal microbiota transplant	Fragmentation Retrieval Polypectomy Instillation
IBD surgery-associated complications	Bleeding control Post-operative ileus Sinusotomy Fistulotomy Clipping or suturing
Colitis-associated neoplasia	Polypectomy Endoscopic mucosal resection Endoscopic submucosal dissection

## Funding

None.
